# A social network analysis on clinical education of diabetic foot

**DOI:** 10.1186/2251-6581-12-44

**Published:** 2013-09-20

**Authors:** Mostafa Shokoohi, Saharnaz Nedjat, Reza Majdzadeh

**Affiliations:** 1 Research Center for Modeling in Health, Institute for Futures Studies in Health, Kerman University of Medical Sciences, Kerman, Iran; 2 School of Public Health, and Knowledge Utilization Research Center (KURC), Tehran University of Medical Sciences, #12, Nosrat East North Kargar, Tehran, Iran; 3 Endocrinology and Metabolism Research Institute, Tehran University of Medical Sciences, Tehran, Iran

## Abstract

**Introduction:**

Identification of Educational Influentials (EIs) in clinical settings helps considerably to knowledge transfer among health and medical practice providers. The aim of this study was identifying EIs in diabetic foot ulcers (DFU) by medical students (clerks, interns and residents) and providing their relational pattern in this subject.

**Methods:**

Subjects were medical students at clerk, intern and resident levels in a local educational hospital. A standard questionnaire with four domains (knowledge, communication, participation and professional ethics) was used for identifying EIs. Students introduced those people with these characteristics who referred them for DFU. Respective communication networks were drawn as intra-group (such as resident-resident) and inter-group (such as intern-resident) networks and quantitative criteria of density, in-degree and out-degree centrality and reciprocity were measured.

**Results:**

The network density of clerks-residents (0.024) and interns-residents (0.038) were higher than clerks-attends (0.015) and interns-attends (0.05); indicating that there were more consulting interactions in former networks than the latter. Degree centrality in residents-related networks (clerks-residents = 2.3; interns-residents = 2.6) were higher than attends-related ones (clerks-attends = 1.1; interns-attends = 1.7), while they were not statistically significant. However, In-degree centralization, which indicating a degree of variance of the whole network of ingoing relationships, in attends-related networks was greater than resident-related networks.

**Conclusion:**

Resident were consulted with almost as same as attends on DFU. It showed that residents were playing a remarkable role in knowledge transfer and they can be considered as EIs in this clinical setting. It seemed that the availability was the main reason for this key role.

## Introduction

There are more than 3 million people over 20 years old with diabetes in Iran. One of the major complications is diabetic foot ulcers (DFUs) and the sever consequence is foot amputation. Disability Adjusted Lost Years (DALY) of type 2 diabetes in Iran was estimated at 4.7 years per 1000 [1] and burden of diabetic foot ulcers and organ amputation were assessed to 5848 and 1573 lost years, respectively [2]. In 2002, approximately 100,000 deaths occurred in Iran due to diabetes [3]. The United Kingdom prospective diabetes study (UKPDS) indicated that 20% of diabetic people suffer from diabetic neuropathy within 4 years after diagnosis of diabetes, which reaches to 50% after 15 years [4]. Lavery et al. in a study on Mexican–Americans and non-Hispanic Whites found that foot amputation is 5.9 per 1,000 ones annually [5].

Health care providers and medical staff including physicians and nurses are the key groups, which could prevent deteriorating problems of DFUs by providing appropriate health care for the patients [6-8]. Studies in Iran have shown that the knowledge, attitude and practice of physicians in the field of diabetes care, especially in the fields of patients“ education, treatment and care of complications, was insufficient [9]. Even studies in developed countries including USA and Netherlands have shown that 30-40% of the patients did not receive health care based on best available evidence, and about 20-25% of the provided cares were insufficient or even potentially harmful [10-12].

Educational Influential (EI) concept and its importance have been introduced to medical education few decades ago. Hiss et al. were among the first people who found the importance of this issue and developed the method for identification of EI in the medical field [13]. Various studies have shown that these people were distinct from other colleagues due to their specific characteristics and had gained an influential and strong role in knowledge transfer and diffusion of innovations. They can lead others to the quality of care and subsequently affect the patients’ health [14-16].

Studies indicated that the most trusted and influential source of information for physicians were their colleagues who communicating with them. Relationships between individuals within a network is as an important source for clinical knowledge and decision making [17,18]. This knowledge significantly influence decision making and management of the patients [19-21]. Pathman et al. proposed a process for implementation of guidelines including awareness, intellectual agreement, decision, adoption and adherence to the guideline or its integration in the routine practice [22]. Traditional continuous medical education (CME) programs generally stopped in the level of awareness or agreement so that they did not reach to the adopt stage. Thus, nowadays considering the adoption stage is an important target in the CMEs. EIs are those people who influence on others’ opinions, attitudes, beliefs, motivations, judgments and behaviors. They can affect adoption of innovation by other practitioners. Knowledge transfer would be easier by identification of these people since in this way, knowledge reached more effectively to the recipients [23].

One way to determine influential individuals within a network, e.g. a hospital, is the social network analysis (SNA). The interpersonal relationships and knowledge transfer patterns within networks can be investigated by using this analysis. Social network consists of a set of actors and their relationships [24]. Tindall and Wellman defined the SNA as analyzing the social structure and its impacts [25]. This analysis can be used for studying the influence of networks’ structure on physicians’ attributes, dissemination of medical knowledge and implementation of the guidelines [26-28].

The aim of this study was identifying the health care providers’ social network in a sample hospital in Iran and knowledge transfer relationships regarding DFU. The specific objective was comparing the role of attends and residents in the aforementioned network.

## Methods

### Subjects

This study was conducted in one of the educational hospitals with about 400 beds in one of the medical sciences universities in Iran. The hospital had five wards including general, surgery, pediatrics, obstetrics and dermatology with approximately 80 faculty members. 140 students participated in this study including 70 clerks, 45 interns and 25 residents.

### Questionnaire

Students were asked to introduce whom they were consulted with for solving their problem (questions) on DFU care. Hiss et al. developed a sociometric instrument for identifying EIs for the first time. It included three domains: knowledge ability, communication skills and humanism. However, authors of the present study developed an identical sociometric questionnaire which published elsewhere and compared characteristics of EI in developed and developing countries [29]. In the later questionnaire, which was used in the present study, the EIs can be identified by four criteria including high level of knowledge, communication skills, taking into account of the stakeholders and following professional ethics.

### Analysis

In the present study, the network was assumed as egocentric. It means that when density is to be calculated, ego nodes and their direct relations are ignored and only relationships between other nodes are considered. Four groups of indicators were estimated in the present study including density, centralization (including in-degree and out-degree), degree centrality and Reciprocity (including dyad and arc).

#### *Density*

It is a description of general situation of links or interconnection of points in the network [30]. This indicator shows the ratio of the existing relations between individuals to a maximum number of possible relationships between them which is measured as a value between zero and one. In a network, the more actors exist in the relationship; the network density will be higher [31-33]. Network density may show the information flow among the nodes.

#### *Centralization*

Centralization implies influential people within a network. Network centralization score ranged between zero to one, that is, zero means every network individual have relationship with every other member, and one means that all members are connected to only one individual. In the other words, the centralization implies the degree of asymmetry in the network. When some individuals have more connections than others, it is expected to have a high centralization score. Centralization has two sub measurements in a directed network: 1) *In*-*degree centralization*: degree of variance of the total network of ingoing relationships in comparison with an ideal star network (which, theoretically, implies the most centralization). Centralization provides an analysis of centrality of consulted individuals. If it is close to one, it means that few individuals of the network are consulted by the rest of the individuals. 2) *Out*-*degree centralization*: degree of variance of the total network of outgoing relationships in comparison with an ideal star network. It shows consulted individuals’ situation, and its high level indicates that small individuals of the network do most of consulting of others [31,33].

#### *Degree centrality*

The mean number of (in/out) going relationships of a person. People who receive many links may be prominent, while subjects who have many connections to others may be influential. The measure refers to direct connections to an individual only [34].

#### *Reciprocity*

Which indicating relationship or interaction between the two individuals symmetrically, that is, if actor A asks consultation from actor B, if actor B asks consultation from actor A too [33]. This indicator was calculated in two ways: 1) *dyad* (or *hybrid*); which means there are several pairs or reciprocated relationship between the actors. This method is obtained from the number of pairs of communication between two individuals within the network divided by existing communications in the network. 2) The *arc* is the other one; i.e. instead of focusing on the number of existing communications, it is focused on the maximum possible number of communications within the network, by focusing on all possible communications within the network.

All of these indicators were calculated for three kinds of communication networks in the following subgroups: A) *Intergroup Communication Networks*: including six subgroups on 1. Clerks with interns, 2. Clerks with residents, 3. Clerks with attends, 4. Interns with residents, 5. Night interns with attends, and 6. Residents with attends. B) *Intra*-*group Communication Networks*: including three subgroups on 1. Clerks with clerks, 2. Interns with interns, and 3. Residents with residents.

UCInet 6 software for windows was used for determining the centrality criteria, providing maps for indicating interpersonal relationships and visual identification of EIs as well as data analysis. NetDraw 2.41 (Network Visualization Software) (a UCInet subprogram) was used for drawing network diagrams [35,36].

The present study was approved by the ethics committee of Tehran University of Medical Sciences (TUMS). An informed consent was obtained from all students who participated to the study.

## Results

**Figure 1:**
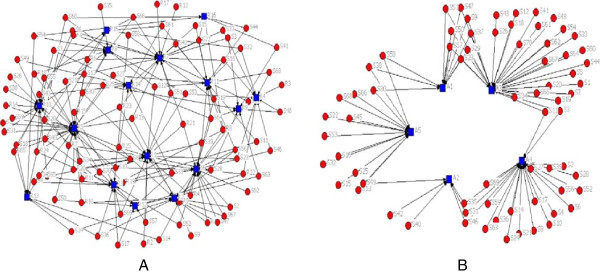
**Clerks**-**residents network (A) vs. clerks-attends network (B).**

**Figure 2:**
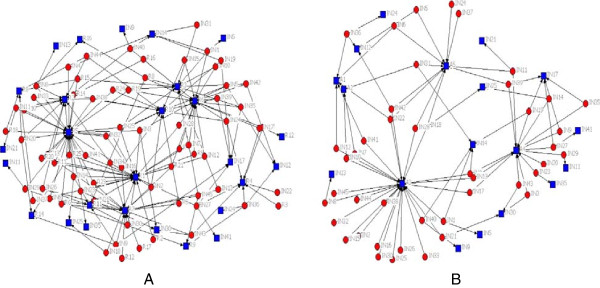
**Interns**-**residents network (A) vs. interns-attends network (B).**

**Figure 3:**
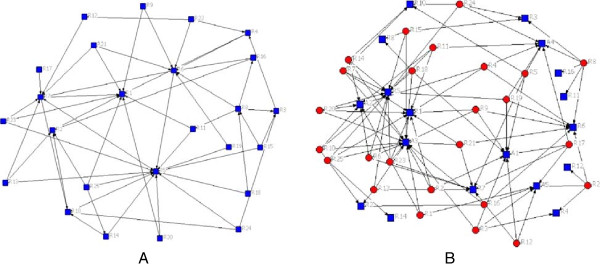
**Residents**-**residents network (A) and residents-attends network (B).**

Figures 1, 2 and 3 present both intergroup and intra-groups connections of the networks. As shown in Figure 1, residents and attends were similarly introduced by clerk as the source of consultation on the DFU. Figure 2 displays connections between interns with residents (2A) and attends (2B). Interns introduced both attends and residents as EIs people. Figure 3A illustrates the relationships within residents when they were seeking for getting the consultation on DFU, it means that residents were not only consulting to the other students, including clerks and interns, but also they were seeking information about DFU from other residents (peers), in addition to the attends (Figure 3B).

Table 1 shows quantitative attributes of the “Intergroup Communication Networks” according to the different subgroups of students. In-degree centralization of the network in attends-related subgroups was not much higher than resident-related subgroups. This indicator for clerks-attends and interns-attends were 42.2% and 52.6%, while for clerks-residents and interns-residents were 31.9% and 38.7%. There were not also considerable differences between attends- and residents-related networks in terms of out-degree centralization of network, which in clerks-attends and clerks-residents, it was 1.1% and 1.8%, and for intern-attends and interns-residents networks equal to 2.6% and 2.0%. However, in- and out-degree centralization belonged to the network of residents-attends subgroups was 45.3 and 6.1%, respectively. Clerks-interns network had the least score in Table 1.

**Table 1:** **Description of inter**-**subgroup networks**

	**Inter subgroups networks**					
	**Clerks with interns**	**Clerks with residents**	**Clerks with attends**	**Interns with residents**	**Interns with attends**	**Residents with attends**
In-degree centralization of network^*^	7.44%	31.9%	42.25%	38.7%	52.6%	45.3%
In-degree centrality, mean ± SD (min, max)	0.58 ± 1.74 (0, 9) ^*^	2.3 ± 5.9 (0, 32)	1.1 ± 4.9 (0, 32)	2.6 ± 5.9 (0, 29)	1.7 ± 4.5 (0, 27)	3.3 ± 4.1 (0, 16)
Out-degree centralization of network	1.25%	1.79%	1.16%	2.01%	2.62%	6.1%
Out-degree centrality, mean ± SD (min, max)	0.58 ± 0.65 (0, 2)	2.3 ± 0.73 (0, 4)	1.1 ± 0.53 (0, 2)	2.6 ± 0.89 (0, 4)	1.7 ± 0.93 (0, 3)	3.3 ± 1.5 (0, 5)
Density	0.005 ± 0.07	0.024 ± 0.15	0.015 ± 0.12	0.038 ± 0.19	0.05 ± 0.18	0.11 ± 0.31

* The definitions are presented in the body text.

The mean degree centrality in resident-related networks was almost higher than attends-related ones. The mean degree centrality for clerks-residents and interns-residents was 2.3 and 2.6, while for clerks-attends and interns-attends was 1.1 and 1.7, respectively. The maximum degree centrality belonged to resident-attends network, while the minimum was to clerks-interns network.

The mean network density of clerks-residents (0.024) and interns-residents (0.038) were higher than that in clerks-attends (0.015) and interns-attends (0.05) networks, indicating that there were more consulting interactions in former networks than the latter. The maximum mean density was related to residents-attends network (0.11 ± 0.31). The reciprocity indicator was zero for all of the subgroups based on both of dyad and arc methods (not showed in the table).

Table 2 shows the “Intra-group Communication Networks” indicators. The estimated indicators for residents-residents communication network were higher compared to other communication networks (interns - interns and clerks - clerks). Values for in-degree, out-degree and density for resident-resident communication network were 46.7%, 7.8% and 0.091, respectively. These three indicators were zero for clerk- clerk communication network. The mean degree centrality in residents-residents and interns-interns networks was 2.2 ± 0.98 and 0.57 ± 0.69, respectively. Reciprocity indicator for resident-resident subgroup was 3.7% and 7.2% according to the dyad and arc.

**Table 2:** **Description of intra**-**subgroup networks**

	**Intra subgroups networks**		
	**Residents with residents**	**Interns with interns**	**Clerks with clerks**
In-degree centralization of network (%)	46.8%	10.2%	0%
In-degree centrality (Mean ± SD) (min, max)	2.2 ± 3.3 (0, 13)	0.57 ± 1.2 (0, 5)	0.0 ± 0.0 (0, 0)
Out-degree centralization of network (%)	7.8%	3.3%	0%
Out-degree centrality (Mean ± SD) (min, max)	2.2 ± 0.98 (0, 4)	0.57 ± 0.69 (0, 2)	0.0 ± 0.0 (0, 0)
Density	0.091 ± 0.28	0.013 ± 0.11	0.0 ± 0.0
Reciprocity			
Dyad or hybrid	3.7%	0%	0%
Arc	7.2%	0%	0%

## Discussion

This study indicated that the communication degree and the distribution of connections in residents-related networks and attends- related ones were nearly similar to each other. In the other words, not only the residents were in relationship with each other, but also the other subgroups including interns and clerks communicated with the residents. This relationship was nearly similar to the communications that the clerks and interns had with their attending.

There are four approaches for identifying EIs: sociometric methods, key informant methods, self-designing methods and observation methods. Most of the studies for identification of EIs used the sociometric instrument, which we used in the present study. A recent systematic review showed that nine trails out of twelve clinical trials used this method [37].

Relatively low density of these networks may be attributed to the type of network, because when density was measured in egocentric method (unlike density measurement based on socio-centric method), ego nodes (those who were consulted with and communicated with other nodes) were not considered. This characteristic of ego-centric network was regarded as a privilege in SNA methods since it sought for finding relationship of other network individuals with each other. In a qualitative study aiming at identification of obstacles in using evidence based medicine and fail to answer their clinical questions, it was shown that several factors including shortage of enough time, lack of access to electronic resources, not having skills required to search electronic resources, lack of tracking unanswered questions, lack of priority for some questions and lack of personal initiative in tracking questions were the barriers. More importantly, team dynamics and institutional culture were considered as the main obstacles. It was indicated that dynamism or team work among medical students especially residents and hospital dominant culture could considerably help transfer of knowledge [38]. In the present study, one reason for justifying low density in communication subgroups, especially in resident - attend and resident - resident subgroups, might be due to the fact that this group of students searched sources other than their colleagues (books, papers, …) for finding the answers for their questions related to the complicated cases such as DFU.

Rappolt performed a qualitative study on 45 family physicians in order to identify sources of clinical information gathering and found that the first source was informal consultants with peers (44%) due to easy accessibility and approachability of them. The second and the third sources for gathering clinical information were asking peer experts (24%) and searching related literature (22%), respectively [17]. In addition, it could be mentioned that the responsibility burden on residents was often higher than the other groups which could justify high communication of this group with others in comparison to other networks.

Reciprocity could show communications among the residents. According to arc indicator, it was 7.2%, implying interaction between the individuals within resident - resident network. However, this indicator was zero for intern - intern and clerk - clerk subgroups, showing lack of interaction among these subgroups. It was clear that the later groups were at the initial states of service provision and possible clinical stresses did not allow them to take risk and rely on each other. However, the residents, who were at higher levels of clinical decision making, consulted with each other. Thus, experience can be considered as an important factor in seeking for consultation from colleagues.

In a survey study conducted at a teaching hospital in Boston, Keating et al. showed that the clinical information had a directed from highly experienced colleagues and accessible colleagues based on the location and schedule. Other predictors to take the clinical consultations among those physicians was having the same gender [39].

Considering in-degree centrality in clerk - attend, clerk - resident, intern - resident, and intern - attend, resident, it is clear that not only the attends could be the consultation source, but also the residents played the role of EI in this setting. High values for these indicators, whether in average or in the range level, suggested the existence of influential individuals within the related networks.

Flodgren et al. in a systematic review found that EIs independently or in conjunction with other interventions could improve evidence-based practice successfully. Effectiveness varied within and between the studies due to high heterogeneities of the collected studies. Therefore, there was no clear way to show and optimize the effectiveness of EIs [40].

Network approaches and science can provide some concepts and techniques to scrutinize the connections between people in any system. Although, network approaches’ application in medical care research is fairly new, in many scientific disciplines such as neurosciences, molecular life sciences, and public health, it has been used. Recent research including studies of patient care teams in treatment of diabetes and chronic heart failure in primary care [32], opinion networks of long-term care specialists [41], and connectedness of health care professionals in the treatment of Parkinson [33]. A social network approach may be particularly relevant if actors (people) have flawed knowledge on their performance options and the disease-related outcomes.

## Competing interests

The authors declare that they have no competing interests.

## Authors’ contributions

Research concept: RM, MS; developing the design: RM, MS and SN; data gathering: MS; analyzing the data: RM, MS and SN, reporting and preparing the manuscript: RM, MS and SN. All authors read and approved the final manuscript.
